# Psychometric evaluation of the Indolent Systemic Mastocytosis Symptom Assessment Form (ISM-SAF) in a phase 2 clinical study

**DOI:** 10.1186/s13023-021-02037-3

**Published:** 2021-10-18

**Authors:** Brad Padilla, Alan L. Shields, Fiona Taylor, Xiaoran Li, Jeffrey Mcdonald, Tanya Green, Anthony L. Boral, Hui-Min Lin, Cem Akin, Frank Siebenhaar, Brenton Mar

**Affiliations:** 1 Adelphi Values, 290 Congress Street 6th Floor, Boston, MA 02210 USA; 2 Blueprint Medicines, Cambridge, MA USA; 3 University of Michigan, Ann Arbor, MI USA; 4 Dermatological Allergology, Department of Dermatology and Allergy, Charité - Universitätsmedizin Berlin, Corporate Member of Freie Universität Berlin, Humboldt-Universität Zu Berlin, and Berlin Institute of Health, Berlin, Germany

**Keywords:** Psychometric evaluation, Instrument development, Patient-reported outcomes, Indolent systemic mastocytosis

## Abstract

**Background:**

Indolent systemic mastocytosis (ISM) is a rare, clonal mast cell neoplasm characterized by severe, unpredictable symptoms. The Indolent Systemic Mastocytosis Symptom Assessment Form (ISM-SAF) items compose a Total Symptom Score (TSS), Gastrointestinal Symptom Score (GSS), and Skin Symptom Score (SSS) to assess symptom severity. This study evaluated the psychometric performance of ISM-SAF among ISM patients.

**Methods:**

In PIONEER, a Phase 2 trial evaluating safety and efficacy of selective kinase inhibitor avapritinib in patients with ISM, the 12-item ISM-SAF was administered daily. Psychometric evaluation of score reliability, validity, and clinical interpretation was conducted using the trial data.

**Results:**

Thirty-eight patients contributed to analyses (78.9% female; mean age = 49). Baseline internal consistency reliability (α) for bi-weekly TSS, GSS, and SSS was 0.86, 0.83, and 0.82, respectively. Test–retest reliability among patients exhibiting no change in Patient Global Impression of Symptom Severity (PGIS) between Baseline and Day 15 exceeded 0.74 universally. Construct validity and known-groups analysis showed moderate to strong ISM-SAF score correlation (r = 0.382–0.881) to supportive patient-reported questionnaires (e.g., PGIS and Mastocytosis Quality of Life Questionnaire) symptom and skin scores, and ability to distinguish among clinically unique groups. Correlations of ISM-SAF and other assessment change scores reflect evidence of score sensitivity. Clinically important difference and response estimates were 7–10 and 19, respectively.

**Discussion:**

ISM-SAF produced reliable, construct-valid, sensitive scores when administered in PIONEER to patients in the target population. Results of this study support the use of the ISM-SAF as a reliable and valid measure to evaluate disease symptomology in ISM patients.

*Trial registration* ClinicalTrials.gov, NCT03731260. Registered 10 October 2018, https://clinicaltrials.gov/ct2/show/study/NCT03731260.

**Supplementary Information:**

The online version contains supplementary material available at 10.1186/s13023-021-02037-3.

## Introduction

Systemic mastocytosis is a rare, clonal mast cell neoplasm driven by the KIT D816V mutation [1], characterized by uncontrolled proliferation and activation of mast cells that leads to severe and unpredictable symptoms for patients with systemic mastocytosis [2]. The incidence of all systemic mastocytosis subtypes is approximately 0.89 per 100,000 per year [3] and the prevalence of indolent systemic mastocytosis (ISM) is estimated at 9.59/100,000 [3]. Many ISM patients experience severe, life-limiting symptoms that significantly impact daily life (e.g., psychological symptoms, neurological symptoms, asthenia) [4, 5]. Currently, there are limited treatment options available for patients with systemic mastocytosis and no approved therapies for patients with ISM [6].

There is a lack of well-defined and reliable measures of disease symptomology to assess the potential clinical benefits of novel treatments for ISM. To address this gap, the Indolent Systemic Mastocytosis Symptom Assessment Form (ISM-SAF) (©2018 Blueprint Medicines Corporation) was developed in ways consistent with regulatory [7] and scientific guidelines [8, 9] to evaluate clinical benefit hypotheses for use in product approval and labeling decisions. The content validity of the ISM-SAF was established using qualitative research methods, along with feedback from regulatory authorities to ensure the ISM-SAF aligned with regulatory expectations for instruments intended for use in clinical trials. Preliminary psychometric evaluation data generated from an observational study supported the trustworthiness of ISM-SAF scores [10], although the interpretation of scores has not yet been evaluated.

The goals of the present study were to psychometrically evaluate the scores produced by the ISM-SAF among ISM patients and inform the interpretation of ISM-SAF scores. Measurement-focused analyses were executed based on blinded data from Part 1 of the Phase 2 PIONEER trial to evaluate the performance of scores produced by the ISM-SAF with respect to score variability, distribution, and missingness; reliability; construct-related validity; and sensitivity to change. Additionally, distribution-based and anchor-based methods were employed to characterize how meaning is attributed to observed ISM-SAF change scores.

## Method

### Study design

**Fig. 1:**
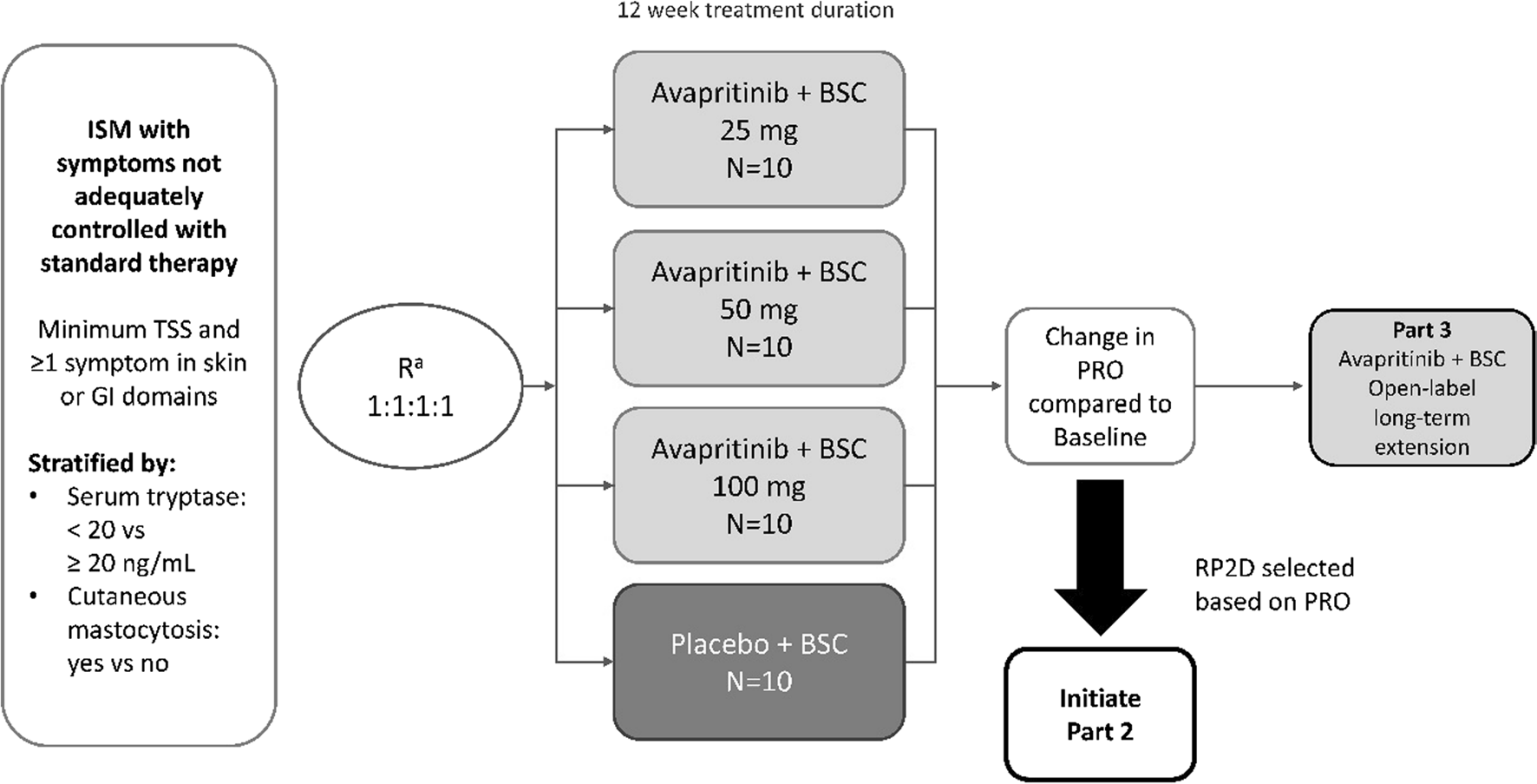
BLU-285-2203 Part 1 study design. *BSC* best supportive care, *GI* gastrointestinal, *ISM* indolent systemic mastocytosis, *PRO* patient-reported outcome, *RP2D* recommended phase 2 dose, *TSS* total symptom score. ^a^All subjects were randomized at the beginning of the study to one of three avapritinib doses or placebo in Part 1

The ISM-SAF was administered daily to patients with ISM enrolled in Part 1 of PIONEER (NCT03731260), a multicenter, randomized, double-blind, placebo-controlled Phase 2 clinical trial to evaluate the safety and efficacy of avapritinib, a potent and selective inhibitor of KIT D816V, in patients with ISM with symptoms inadequately controlled with standard therapy (Fig. 1).

### Analysis populations

Two analysis populations were defined: (1) a cross-sectional analysis population (CS-AP) composed of all patients with at least one response on the ISM-SAF evaluated at Baseline (biweekly period from Cycle 1 Day-14 [C1D-14] to C1D-1) and at least one biweekly follow-up score at either Cycle 3 (C3D-14 to C3D-1) or Cycle 4 (C4D-14 to C4D-1); and (2) a test–retest analysis population (TRT-AP) composed of patients who exhibited no change in Patient Global Impression of Severity (PGIS) score from Baseline to C1D15 who provided at least one response for the ISM-SAF at both Baseline and Timepoint 2 (C1D1 to C1D14).

### Study assessments

#### ISM-SAF

The ISM-SAF is a 12-item diary that assesses 11 symptoms of ISM, including bone pain, abdominal pain, headache, nausea, spots, itching, flushing, fatigue, dizziness, brain fog, and diarrhea, over a 24-h period. Eleven items assess symptom severity using an 11-point numeric rating scale, where 0 = No [symptom] and 10 = Worst imaginable [symptom]; the twelfth item measures diarrhea frequency by asking patients to enter a discrete numerical value. Developed in United States English, the ISM-SAF underwent translation and linguistic/cultural validation in all relevant languages prior to implementation in PIONEER. A handheld electronic device was used to administer the ISM-SAF daily.

The ISM-SAF is scored as a 14-day average at the item, domain, and total score levels. The two symptom domains include the Gastrointestinal Symptom Score (GSS), composed of abdominal pain, nausea, and diarrhea severity (score range 0–30), and the Skin Symptom Score (SSS), composed of spots, itching, and flushing severity (score range 0–30). The Total Symptom Score (TSS) is composed of all 11 severity items (range 0–110). The daily domain and total scores are generated by summing the item scores for contributing items each day; if any contributing items are missing for the day, the daily score cannot be calculated. Biweekly scores were derived by averaging scores over 14 days, with a minimum of seven daily scores required.

#### Supportive measures

Psychometric evaluation of the ISM-SAF was supported by other patient-reported outcome (PRO) assessments, which were administered at Baseline (except for the Patient Global Impression of Change [PGIC]), C3D1, and C4D1. The administration of the Patient Global Impression of Severity (PGIS) at C1D15 was also used to evaluate test–retest reliability.

##### 12-Item Short Form Health Survey (SF-12v2®)

The SF-12v2® is a 12-item PRO questionnaire developed for a general population assessing physical and emotional health and function using a recall period of “the past week” on three- and five-point verbal response scales (scores range from 0 to 100, with higher scores representing better health) [11, 12].

##### Mastocytosis Quality of Life Questionnaire (MC-QoL)

The MC-QoL is a 27-item PRO questionnaire assessing the domains of symptoms, emotions, social life/functioning, and skin in patients with cutaneous mastocytosis and ISM [13]. The questionnaire uses a recall period of “the past two weeks” and a five-point verbal rating scale (scores ranges from 0 to 100, where higher scores indicate higher health-related quality-of-life impairment).

##### Patient Global Impression of Severity (PGIS)

The PGIS is a single item that asks patients to rate their overall symptom severity “right now” on a five-point scale (“0—absent,” “1—minimal,” “2—moderate,” “3—severe,” and “4—very severe”).

##### Patient Global Impression of Change (PGIC)

The PGIC item assesses a patient’s perception of the change in the state of their condition at a point in time (“degree of change since beginning care at this clinic”) on an 11-point numeric rating scale measuring the full spectrum of change (0 = much better, 5 = no change, and 10 = much worse).

##### Five-level EQ-5D (EQ-5D-5L)

The EQ-5D-5L is used to measure current health status and provide a generic measure of health for clinical assessment. It comprises two parts: the EQ-5D-5L descriptive system and the EQ-5D-5L Visual Analogue Scale (VAS). The EQ-5D-5L VAS is a single item that asks respondents to self-rate their health on a VAS ranging from 0 to 100 where lower scores indicate a lower overall health state. Only the EQ-5D-5L VAS contributed to the psychometric analyses in this study.

### Analyses

All analyses were conducted in SAS 9.4 and focused on evaluating the performance of the ISM-SAF. There was no imputation of missing data. Unless otherwise specified, analyses were conducted using data at C1D1, C3D1, and C4D1, with C1D15 data additionally used to evaluate test–retest reliability.

#### Study sample

Descriptive statistics for age, sex, and race were computed for the study sample using the data generated from the CS-AP at Baseline.

#### Score distribution

Item-level and domain-level ISM-SAF score distributions were evaluated in terms of respondents’ use of the entire scale and for floor and ceiling effects.

#### Inter-item correlations

Inter-item correlations were evaluated to characterize the extent to which scores on one item of the ISM-SAF relate to scores produced by the other items within that same multi-item scale/domain. Guidelines used to facilitate interpretation of correlations were as follows: negligible relationship, r = 0.0–0.09; small relationship, r = 0.1–0.29; medium relationship, r = 0.30–0.49; and strong relationship, r ≥ 0.50 [14, 15].

#### Reliability

Reliability estimates characterize consistency and reproducibility of a particular set of scores produced by a questionnaire when administered to a particular target patient population and in a particular context of use [16]. In this study, the reliability of the ISM-SAF was investigated in terms of both internal consistency reliability and test–retest reliability. Internal consistency reliability, which reflects to what extent individual items are measuring the same general concept [17], was investigated by calculating Cronbach’s alpha coefficient (α, range 0 to 1). Alpha was calculated for the biweekly TSS, GSS, and SSS using the CS-AP at Baseline, C3D1, and C4D1 and again with each individual item within a domain removed. Scores greater than 0.70 are typically seen as sufficient for research purposes [18]. Test–retest reliability, which assesses whether items produce stable scores at different assessment points during which no change (or minimal change) in the patient’s condition is expected to occur [19], was evaluated in the TRT-AP using ISM-SAF biweekly scores at Baseline and C1D15. Intra-class correlation coefficients (ICCs) greater than 0.70 are evidence of adequate test–retest reliability [20].

#### Validity

Construct-related validity measures the associations between concepts of a specified assessment and of other assessments (i.e., reasonably strong associations should exist between related concepts, and low associations between unrelated concepts), and was evaluated for the biweekly ISM-SAF scores by generating correlation coefficients between its scores and other PRO assessments at Baseline, C3D1, and C4D1. The same guidelines were used to facilitate interpretation of correlations as for inter-item correlations.

Known-groups methods characterize the degree to which a PRO questionnaire generates scores capable of distinguishing among patient groups hypothesized to be clinically distinct [7]. This analysis was conducted using the PGIS, EQ-5D-5L VAS, MC-QoL, and SF-12v2® to categorize patients into “known groups” at Baseline, and ISM-SAF biweekly scores were described across patient severity groups. It was hypothesized that higher ISM-SAF scores (greater symptoms) would be associated with worse symptoms/quality of life scores on the other instruments.

#### Sensitivity to change

Sensitivity-to-change analyses were conducted by examining the mean change and associated effect size [14] of biweekly ISM-SAF scores, as well as the correlations between the ISM-SAF change scores and change scores of other measures. It was hypothesized that improvements (or worsening) in ISM-SAF scores would correspond to improvements (or worsening) in other related measures.

#### Interpretation of scores

Score interpretation analysis informs how meaning is attributed to the change detected by a PRO questionnaire. Distribution-based methods utilize the observed distribution of the data to generate clinically important difference (CID) estimates, or the difference in mean scores between two treatment groups that can be considered clinically relevant [21, 22]. Two distribution-based analyses were employed here for the biweekly ISM-SAF scores: (1) ½ standard deviation (SD) at Baseline and (2) standard error of measurement (SEm). Anchor-based methods use external criteria (PGIS) to categorize patients into groups, each reflecting an a priori-determined change grouping (e.g., no change, positive change, or negative change), and were employed to generate clinically important response (CIR) estimates to inform conclusions about the meaning of observed within-person change in the scores of the ISM-SAF [22, 23].

## Results

### Study sample

A total of 38 eligible patients contributed to the psychometric-focused analysis, with < 3% (n = 1) of patients having missing biweekly severity item scores at C3D1 and C4D1. The average age of the CS-AP cohort was 49.0 years (SD = 13), 78.9% of the patients were female (n = 30), and 92.1% of the patients were White (n = 35). Complete demographic and health details are presented in Additional file 1: Table S1.

### Score distribution

Descriptive analysis of the ISM-SAF indicated that, while patients used the range of response options available to them for each item (i.e., 0 to 10), not all patients reported experiencing all symptoms and, when symptoms were reported, severity rates were variable. The mean scores of severity items ranged from 3.0 (diarrhea) to 7.2 (fatigue); the mean TSS, GSS, and SSS were 54.2, 10.9, and 16.2, respectively, at Baseline.

### Inter-item correlations

At Baseline, the GSS items (abdominal pain, nausea, and diarrhea) were moderately to strongly correlated with one another (r = 0.46 to 0.83), while the SSS items (spots, itching, and flushing) were also moderately to strongly correlated with one another (r = 0.46 to 0.76). The GSS items and other symptom severity items (bone pain, fatigue, dizziness, brain fog, and headache) were moderately to strongly correlated with one another at Baseline (r = 0.41 to 0.67) with the exception of abdominal pain and nausea with bone pain (r = 0.28), and diarrhea severity with headache (r = 0.13). The SSS items and other symptom severity items had small to medium relationships at Baseline (r = 0.11 to 0.42) with the exception of the spots item, which had negative and negligible to small relationships with other symptom items (r = -0.26 to -0.07). In addition, the SSS items were negligibly to moderately related to the GSS items (r = -0.02 to 0.44). As expected, results indicated a strong relationship between the diarrhea frequency and severity items (r = 0.72 at Baseline). As a wider range of values were available for the ISM-SAF at C3D1 and C4D1, the correlations among items were generally enhanced at the later timepoints.

### Reliability

#### Internal consistency reliability

Internal consistency estimates (α) for the TSS, GSS, and SSS biweekly scores are presented in Table 1 and exceeded pre-specified criteria for adequate reliability (α ranged from 0.72 to 0.86). Removal of items from the TSS did not result in an appreciable increase in alpha coefficients; removal of the diarrhea severity item and spots item resulted in an increase in the Cronbach’s alpha for the GSS and SSS, respectively.

**Table 1:** Internal consistency reliability estimates of biweekly ISM-SAF domain and total scores

Domain/total score	Cronbach's alpha		
	Baseline N = 38	C3D1 N = 37	C4D1 N = 36
Total symptom score	0.86	0.85	0.86
*Alpha if item deleted*			
Q1. Bone pain	0.84	0.84	0.84
Q2. Abdominal pain	0.83	0.83	0.84
Q3. Nausea	0.84	0.83	0.84
Q4. Spots	0.88	0.88	0.89
Q5. Itching	0.84	0.83	0.84
Q6. Flushing	0.85	0.84	0.85
Q7. Fatigue	0.84	0.83	0.84
Q8. Dizziness	0.83	0.83	0.84
Q9. Brain fog	0.84	0.83	0.84
Q10.Headache	0.85	0.84	0.85
Q12. Diarrhea severity	0.84	0.85	0.86
Gastrointestinal Symptom Score	0.83	0.73	0.72
*Alpha if item deleted*			
Q2. Abdominal pain	0.63	0.37	0.44
Q3. Nausea	0.73	0.55	0.44
Q12. Diarrhea severity	0.91	0.88	0.90
Skin Symptom Score	0.82	0.76	0.76
*Alpha if item deleted*			
Q4. Spots	0.87	0.79	0.83
Q5. Itching	0.63	0.61	0.52
Q6. Flushing	0.73	0.63	0.64

*C#D#* cycle number, day number, *ISM-SAF* Indolent Systemic Mastocytosis Symptom Assessment Form

### Test–retest reliability

Test–retest reliability ICCs for the biweekly ISM-SAF TSS, GSS, SSS, and item scores for patients maintaining the same PGIS rating at Baseline (C1D1) and at C1D15 (as their scores are expected to remain stable) are presented in Table 2. All ICCs exceeded 0.7 (ranged from 0.741 to 0.986), indicating that the biweekly item, domain, and total ISM-SAF scores were all reliable.

**Table 2:** Test–retest reliability between baseline and C1D15 (n = 17)

ISM-SAF items/domains	n	ICC (95% confidence interval)^a^
Total Symptom Score	16	0.956
Gastrointestinal Symptom Score	16	0.858
Skin Symptom Score	17	0.981
Q1. Bone pain	17	0.867
Q2. Abdominal pain	17	0.935
Q3. Nausea	17	0.858
Q4. Spots	17	0.986
Q5. Itching	17	0.949
Q6. Flushing	17	0.959
Q7. Fatigue	17	0.878
Q8. Dizziness	17	0.936
Q9. Brain fog	17	0.917
Q10. Headache	17	0.944
Q11. Diarrhea frequency	17	0.932
Q12. Diarrhea severity	16	0.741

*C#D#* cycle number, day number, *ICC* intraclass correlation coefficient, *ISM-SAF* Indolent Systemic Mastocytosis Symptom Assessment Form

^a^The ICC was computed using the single measurement, absolute agreement, two-way mixed effects model

### Validity

#### Construct-related validity

The relationships between the TSS and other variables were strong and in the expected direction. Specifically, at C4D1, the biweekly ISM-SAF domain and total scores were more strongly correlated (r = 0.382 to 0.881) to the PGIS, MC-QoL symptom and skin scores than to more distal concepts. Correlations with other measures were generally greater for the TSS than for the GSS and SSS, except for the MC-QoL skin domain, which correlated most strongly with the SSS as expected (Table 3).

**Table 3:** Pearson correlations of ISM-SAF total and domain scores with other measures administered at C4D1 (N = 34^a^)

Concurrent scores	Pearson correlation^b^		
	Total symptom score	Gastrointestinal symptom score	Skin symptom score
*SF-12v2®*			
Physical functioning	− 0.390	− 0.329	− 0.212
Role-physical	− 0.566	− 0.357	− 0.348
Bodily pain	− 0.459	− 0.421	− 0.056
General health	− 0.558	− 0.363	− 0.306
Vitality	− 0.518	− 0.354	− 0.170
Social functioning	− 0.358	− 0.365	− 0.275
Role-emotional	− 0.255	− 0.329	− 0.166
Mental health	− 0.215	− 0.216	− 0.178
MCS	− 0.226	− 0.275	− 0.179
PCS	− 0.536	− 0.368	− 0.218
*MC-QoL*			
Symptoms	0.695	0.541	0.382
Social life/functioning	0.624	0.498	0.498
Emotions	0.456	0.399	0.329
Skin	0.530	0.265	0.881
Total score	0.695	0.538	0.550
*EQ-5D-5L*			
EQ-5D-5L VAS	− 0.612	− 0.432	− 0.328
*PGIS*			
PGIS	0.656[a]	0.401[a]	0.618[a]

ISM-SAF biweekly item scores range from 0 to 10 with higher scores associated with worse signs and symptoms, with the exception of Item 11, which asks about frequency of events. ISM-SAF biweekly domain scores range from 0 to 30 and total score ranges from 0 to 110 with higher scores associated with more severe signs and symptoms. The SF-12v2® scores are norm-based normalized to United States general population with mean 50 and standard deviation of 10, with higher score indicating better functioning or well-being. The MC-QoL scores range from 0 to 100 where higher scores represent more health-related quality of life impairment. The EQ-5D-5L VAS ranges from 0 to 100 where higher scores represent better health states

*C#D#* cycle number, day number, *EQ-5D-5L* five-level EQ-5D, *ISM-SAF* Indolent Systemic Mastocytosis Symptom Assessment Form, *MC-QoL* Mastocytosis Quality of Life Questionnaire, *MCS* Mental Component Summary, *PCS* Physical Component Summary, *PGIS* Patient Global Impression of Severity, *VAS* visual analogue scale

^a^N value is based on number of subjects with both ISM-SAF domain scores and concurrent scores available at Baseline

^b^Correlations and p-values were calculated as Pearson's correlations and Fisher's z-transformation except [a]ordinal variables where correlations and p-values were calculated using polyserial correlations and likelihood ratio tests

#### Known-groups analysis

ISM-SAF TSS scores were able to distinguish among clinically unique groups, as evidenced by clearly distinct scores in the hypothesized direction (i.e., participants with greater symptoms, as assessed by the PGIS, EQ-5D-5L VAS, MC-QoL Symptoms, and SF-12v2® Physical Component Summary (PCS), also scored higher on the ISM-SAF). These differences in scores were statistically significant (*p* < 0.05) across all groups for the TSS at C4D1 (Table 4). For the GSS and SSS, scores for most groups also trended in the hypothesized direction, although the differences were not always significant. In cases where the mean and median scores for GSS and SSS were similar between adjacent severity groups, any deviations from hypotheses were likely due to the limitation of sample size.

**Table 4:** Known-groups analysis of ISM-SAF scores based on concurrent assessments administered at C4D1

ISM-SAF domain	Known group	N	Mean (SD)	Median	*p* value*
*PGIS*					
Gastrointestinal Symptom Score (0–30)	Absent/Minimal	12	4.3 (3.3)	4.2	0.009
	Moderate	11	9.5 (2.9)	9.4	
	Severe/Very Severe	11	9.7 (6.5)	9.4	
Skin Symptom Score (0–30)	Absent/Minimal	12	8.1 (4.0)	8.5	< 0.001
	Moderate	11	12.6 (6.0)	10.9	
	Severe/Very Severe	11	18.3 (6.5)	20.4	
Total Symptom Score (0–110)	Absent/Minimal	12	27.1 (10.2)	26.8	< 0.001
	Moderate	11	50.4 (12.1)	50.9	
	Severe/Very Severe	11	55.2 (15.1)	55.9	
*EQ-5D-5L VAS*					
Gastrointestinal Symptom Score (0–30)	Severe (1st tertile)	11	11.0 (5.6)	10.6	0.028
	Moderate (2nd tertile)	12	6.5 (5.0)	6.2	
	Mild (3rd tertile)	11	5.8 (2.9)	5.1	
Skin Symptom Score (0–30)	Severe (1st tertile)	11	16.2 (5.9)	19.0	0.144
	Moderate (2nd tertile)	12	11.8 (7.9)	9.4	
	Mild (3rd tertile)	11	10.8 (5.9)	9.9	
Total Symptom Score (0–110)	Severe (1st tertile)	11	55.5 (15.7)	55.9	0.003
	Moderate (2nd tertile)	12	44.0 (16.1)	44.1	
	Mild (3rd tertile)	11	31.5 (13.0)	28.1	
*MC-QoL Symptom*					
Gastrointestinal Symptom Score (0–30)	Mild (1st tertile)	10	4.7 (3.6)	4.2	0.014
	Moderate (2nd tertile)	12	7.3 (3.7)	7.6	
	Severe (3rd tertile)	12	10.7 (5.8)	11.1	
Skin Symptom Score (0–30)	Mild (1st tertile)	10	9.7 (7.1)	9.0	0.104
	Moderate (2nd tertile)	12	12.5 (6.3)	10.4	
	Severe (3rd tertile)	12	15.9 (6.5)	19.3	
Total Symptom Score (0–110)	Mild (1st tertile)	10	29.5 (10.6)	27.6	< 0.001
	Moderate (2nd tertile)	12	43.4 (16.4)	46.3	
	Severe (3rd tertile)	12	55.8 (14.8)	55.7	
*SF-12v2® PCS*					
Gastrointestinal Symptom Score (0–30)	Severe (1st tertile)	11	10.6 (3.2)	10.6	0.032
	Moderate (2nd tertile)	12	7.6 (6.5)	5.7	
	Mild (3rd tertile)	11	5.0 (3.3)	4.5	
Skin Symptom Score (0–30)	Severe (1st tertile)	11	13.8 (7.1)	15.0	0.366
	Moderate (2nd tertile)	12	14.2 (5.7)	13.0	
	Mild (3rd tertile)	11	10.4 (7.7)	9.6	
Total Symptom Score (0–110)	Severe (1st tertile)	11	53.4 (10.8)	55.6	0.002
	Moderate (2nd tertile)	12	47.8 (19.6)	45.0	
	Mild (3rd tertile)	11	29.4 (11.4)	27.1	

*C#D#* cycle number, day number, *EQ-5D-5L* Five-level EQ-5D, *ISM-SAF* Indolent Systemic Mastocytosis Symptom Assessment Form, *MC-QoL* Mastocytosis Quality of Life Questionnaire, *PCS* Physical Component Summary, *PGIS* Patient Global Impression of Severity, *SD* standard deviation, *VAS* visual analogue scale

^*^p values from are from one-way analysis of variance (ANOVA) testing of mean score differences between groups

### Sensitivity to change

The results indicated that all ISM-SAF scores were sensitive to change, as shown by a decrease from Baseline to C4D1. The mean change scores of the biweekly TSS (− 12.70 [SD = 14.93]), GSS (− 3.83 [SD = 5.98]), and SSS (− 4.07 [SD = 5.64]) all had a moderate effect size (0.8 > *d* ≥ 0.5). In addition, the results indicated that from Baseline to C4D1, the change scores of the TSS, GSS, and SSS were strongly correlated with each other (r ≥ 0.50) and moderately to strongly correlated with the change scores in the PGIS, EQ-5D-5L VAS, SF-12v2®, MC-QoL domain and total scores, and PGIC (Additional file 1: Table S2), indicating sensitivity to change.

### Interpretation of scores

Candidate between-group CIDs for ISM-SAF biweekly scores were generated using distribution-based methods (TSS = 7–10, GSS = 2–4, and SSS = 3–4 points) (Table 5). Among patients who “improved” (n = 16) based on a PGIS reduction of one or two units, the ISM-SAF biweekly TSS, GSS, and SSS decreased 19.0, 6.4, and 6.2 with an average individual percent decrease of 29.4%, 8.4%, and 36.3% at C4D1 from Baseline, respectively. CID estimates based on changes from Baseline to C3D1 were slightly lower (16.2, 6.0, and 4.7 for the TSS, GSS, and SSS, respectively).

**Table 5:** Distribution-based methods to estimate clinically important difference for ISM-SAF biweekly domain and total scores

ISM-SAF biweekly domain	n	Reliability^a^	1/2 SD	SEm
Total Symptom Score	37	0.86	9.53	7.22
Gastrointestinal Symptom Score	37	0.83	3.48	2.86
Skin Symptom Score	38	0.82	3.76	3.19

ISM-SAF = Indolent Systemic Mastocytosis Symptom Assessment Form; SD = standard deviation, SEm = standard error of measurement (SD at Baseline multiplied by the square root of [1-reliability])

^a^Reliability estimate from Baseline Cronbach’s alpha internal consistency reliability estimates

## Discussion

The results of the psychometric analysis of the TSS scores produced by the ISM-SAF in Part 1 of PIONEER provide evidence of the reliability and validity of the ISM-SAF’s scores and help to inform score interpretation of the ISM-SAF in future clinical studies.

The data showed strong compliance with the ISM-SAF across all timepoints, with only one patient missing a TSS score at C3D1 and C4D1. The ISM-SAF was able to produce reliable scores in terms of internal consistency and test–retest reliability. The biweekly TSS, GSS, and SSS all met the pre-specified criterion for internal consistency (α > 0.70) at Baseline, and the removal of items from TSS did not appreciably increase alpha coefficients. Test–retest reliability exceeded 0.70 for all biweekly scores. The scores produced by the ISM-SAF were also concluded to be construct-valid based on the evidence that they moderately to strongly correlated with other assessments as expected (e.g., PGIS, MC-QoL symptom and skin scores). In addition, as evidence of validity by known-groups analysis, TSS was clearly distinct by PGIS, EQ-5D-5L VAS, MC-QoL symptom, and SF-12v2® PCS score groups in the hypothesized direction. Lastly, the ISM-SAF scores were also observed to be sensitive to change, as shown by all ISM-SAF scores decreasing from Baseline to C4D1, and the moderate to strong correlation of change scores on the ISM-SAF with change scores of other instruments measuring similar concepts.

Candidate between-group CIDs for ISM-SAF bi-weekly scores were generated using distribution-based methods and, based on a range of 7–10 scale units for the TSS, a 10-point threshold was chosen as a conservative approach to provide guidance for interpreting substantive results when using ISM-SAF for the comparison of treatment group mean differences. Candidate CIR estimates were generated using anchor-based methods based on changes in ISM-SAF scores for those patients who improved on the PGIS from Baseline to C3D1 and C4D1. Based on the upper limit of the range of estimates for individual percentage decrease (i.e., 29.4% for TSS using PGIS anchor at C4D1), a 30% individual percentage decrease on the TSS was selected as a conservative estimate to represent the CIR or improvement at the individual level for future efficacy analyses.

There were a few limitations in this study. The removal of the diarrhea item resulted in a notable increase of Cronbach’s alpha for GSS, and the removal of the spots item also resulted in an increase in the alpha coefficient. The decision as to whether an item should be removed from the calculation of a domain or total score is not solely based on the Cronbach’s alpha coefficient, and the conceptual framework of the measure (e.g., the relevance of diarrhea to gastrointestinal key signs and symptoms) generated from patient interviews should be taken into consideration. For example, based on the results from concept elicitation patient interviews, 75% of the patients (n = 12/16) identified diarrhea as a symptom of ISM, and 90% of patients (n = 9/10) cognitively debriefed reported having experienced diarrhea due to their ISM. Therefore, even though 47.4–62.2% of patients (n = 18–23) in Part 1 of PIONEER scored zero (i.e., no diarrhea) at each biweekly assessment timepoint used in analyses, which might affect the internal consistency of GSS, it was not recommended that the diarrhea severity item be removed from the scale.

Additionally, the confidence in the statistical analysis was reduced due to the limited sample size. Although the ISM-SAF TSS was clearly distinct by PGIS, EQ-5D-5L VAS, MC-QoL, and SF-12v2® groups, the small sample size (N = 38 for CS-AP) limited the interpretation of these known-groups analyses (n < 10 for some groups). Additionally, the small to moderate effect sizes generated using these data were expected because the change from Baseline to C4D1 was examined with combined treatment groups and placebo group, given the blinded nature of the data on which these estimates were based. Furthermore, given the limitations of the PGIC version implemented in the study (e.g., not specific to change in symptoms, and potential recall bias), only CIR estimates generated using PGIS anchors are reported here.

The ISM-SAF was developed through qualitative research including both patients with ISM and those with smoldering systemic mastocytosis. Although the psychometric analyses presented here are based on an ISM population, the findings are consistent with preliminary psychometric analyses that were previously conducted through an observational study involving both patients with ISM and those with smoldering systemic mastocytosis [10], thereby supporting the use of the ISM-SAF in this broader population.

In conclusion, the ISM-SAF produced reliable, construct-valid, and sensitive scores when administered in the target patient population participating in a regulated clinical trial, with a CIR definition of a 30% individual percentage decrease on the TSS. These results, along with the ISM-SAF’s strong development history and evidence of content validity, support its use in clinical studies designed to evaluate ISM treatments and impact on patient symptom improvement.

## Supplementary Information

**Additional file 1:** Demographic information and sensitivity to change results. **Table S1.** Sample demographic information at Baseline (N = 38). **Table S2.** Sensitivity to change: Correlation between ISM-SAF biweekly domain and total change scores and change in concurrently administered measures from Baseline to C4D1 (N = 36).

## Data Availability

The datasets generated and/or analyzed during the current study are not publicly available due to the fact that the analyses described in this publication were executed based on blinded data from an ongoing Phase 2 clinical trial but could be available from the corresponding author on reasonable request.

## Abbreviations

CID: Clinically important difference
CIR: Clinically important response
CS-AP: Cross-sectional analysis population
EQ-5D-5L: Five-level EQ-5D
GSS: Gastrointestinal Symptom Score
ICC: Intraclass correlation coefficient
ISM: Indolent systemic mastocytosis
ISM-SAF: Indolent Systemic Mastocytosis Symptom Assessment Form
MC-QoL: Mastocytosis Quality of Life Questionnaire
PGIC: Patient Global Impression of Change
PGIS: Patient Global Impression of Severity
PRO: Patient-reported outcome
SD: Standard deviation
SSS: Skin Symptom Score
TRT-AP: Test–retest analysis population
TSS: Total Symptom Score
VAS: Visual analogue scale
