# Supplementary material for: Psychometric evaluation of the Indolent Systemic Mastocytosis Symptom Assessment Form (ISM-SAF) in a phase 2 clinical study
Source: Orphanet J Rare Dis. 2021 Oct 18;16:434. doi: 10.1186/s13023-021-02037-3 (PMC8522163; doi:10.1186/s13023-021-02037-3)
Supplement: Supplementary file 1 — Additional file 1: Demographic information and sensitivity to change results. Table S1. Sample demographic information at Baseline (N = 38). Table S2. Sensitivity to change: Correlation between ISM-SAF biweekly domain and total change scores and change in concurrently administered measures from Baseline to C4D1 (N = 36). [file 13023_2021_2037_MOESM1_ESM.docx]

Supplementary tables

Supplementary Table 1. Sample demographic information at Baseline (N=38)

| Characteristic | Statistic or n (%) |
| --- | --- |
| Age (years) | |
| N | 38 |
| Mean (SD) | 49.0 (13.0) |
| Median | 50.5 |
| Min-Max | 21.0–75.0 |
| Sex (n, %) | |
| Female | 30 (78.9%) |
| Male | 8 (21.1%) |
| Race (n, %) | |
| White | 35 (92.1%) |
| Black or African American | 1 (2.6%) |
| Unknown | 2 (5.3%) |

Abbreviations: Min=minimum; Max=maximum; SD=standard deviation

Supplementary Table 2. Sensitivity to change: Correlation between ISM-SAF biweekly domain and total change scores and change in concurrently administered measures from Baseline to C4D1 (N=36)

|  | ISM-SAF biweekly domains | |  |
| --- | --- | --- | --- |
|  | Total symptoms score | Gastrointestinal symptom score | Skin symptom score |
| ISM-SAF bi-weekly domains (N=35) | | | |
| Gastrointestinal Symptom Score | 0.861 | 1 | 0.554 |
| Skin Symptom Score | 0.771 | 0.554 | 1 |
| Total Symptom Score | 1 | 0.861 | 0.771 |
| SF-12v2® (N=31) | | | |
| Physical functioning | -0.360 | -0.331 | -0.256 |
| Role-physical | -0.482 | -0.424 | -0.316 |
| Bodily pain | -0.378 | -0.236 | -0.449 |
| General health | -0.512 | -0.323 | -0.494 |
| Vitality | -0.393 | -0.291 | -0.309 |
| Social functioning | -0.681 | -0.526 | -0.689 |
| Role-emotional | -0.306 | -0.213 | -0.433 |
| Mental health | -0.410 | -0.358 | -0.266 |
| MCS | -0.444 | -0.337 | -0.459 |
| PCS | -0.409 | -0.309 | -0.337 |
| MC-QoL (N=31) | | | |
| Symptoms | 0.725 | 0.561 | 0.624 |
| Social life/functioning | 0.566 | 0.390 | 0.553 |
| Emotions | 0.609 | 0.438 | 0.541 |
| Skin | 0.393 | 0.229 | 0.577 |
| Total score | 0.699 | 0.501 | 0.670 |
| EQ-5D-5L (N=31) | | | |
| EQ-5D VAS | -0.580 | -0.467 | -0.532 |
| PGIS (N=31) and PGIC (N=33) | | | |
| PGIS | 0.706 | 0.564 | 0.642 |
| PGIC | 0.557 | 0.314 | 0.634 |

Abbreviations: C#D#=Cycle number, Day number; EQ-5D-5L=Five-level EQ-5D; ISM-SAF=Indolent Systemic Mastocytosis Symptom Assessment Form; MC-QoL=Mastocytosis Quality of Life Questionnaire; MCS=Mental Component Summary; PCS=Physical Component Summary; PGIC=Patient Global Impression of Change; PGIS=Patient Global Impression of Severity; VAS=visual analogue scale
